# Editorial: Innovative educational methods for Family Medicine and Primary Care training

**DOI:** 10.4102/phcfm.v16i1.4833

**Published:** 2024-12-11

**Authors:** Sunanda C. Ray

**Affiliations:** 1 Department of Medical Education, Faculty of Medicine, University of Botswana, Gaborone, Botswana

The *African Journal of Primary Health Care and Family Medicine* put out a call for short reports to showcase innovations in teaching and learning. Twenty of the submissions received have been published in this special collection. The reports came from Angola, Ethiopia, Ghana, Kenya, Malawi, Namibia, South Africa and Zimbabwe. The key themes from these reports are summarised here.

The training of family physicians (FPs) must equip them to perform as clinicians, consultants, clinical trainers, capacity builders, team-leaders, leaders of clinical governance and champions of community-orientated primary care (PC).^1^ The training must also enable them to strengthen district health systems including PC, where they can have the most impact on health outcomes.^2,3^ In South Africa, all specialist training programmes are moving towards workplace-based assessment (WPBA).^3^ Authors described the need to train and assess FPs in the same workplace where they will eventually practise autonomously.^4,5,6^ The development of instruments to conduct WPBA in FP training was explained, with the creation of entrustable professional activities (EPAs) as the specific tasks or responsibilities that registrars are ‘entrusted’ to be able to perform independently by the end of their training.^3,7^ Other developments described in these reports for training FP registrars included training of clinical trainers, blended learning methods, development of e-portfolios, and use of group coaching for leadership and governance skills.^4,7,8,9^ Decentralised or distributed FP learning took place in remote or rural areas of several countries,^9,10^ with virtual co-teaching and faculty development, learnt during the coronavirus disease 2019 (COVID-19) pandemic, integrated into collaborative training programmes.^11^

The importance of including Family Medicine (FM) and PC concepts from early stages of the undergraduate medical curriculum was advocated by several authors, to raise awareness of the principles of FM in providing continuity and coordination of holistic care, managing undifferentiated conditions and responding to the health needs of communities. Familiarity with FM as a speciality is critical to encourage young doctors to consider it as a rewarding career choice.^12^ Many FM or PC placements were in rural areas or urban municipal clinics. Innovative approaches in undergraduate FM training included use of audio-visual aids and case study methods to explore core values of FM;^12^ use of a checklist to appraise the inclusion of Primary Health Care (PHC) concepts in the undergraduate medical curriculum;^13^ and an online module on the Moodle platform with a study guide, an electronic portfolio, and electronic resources (e-books and apps).^14^ Interprofessional training components in PC and faculty development for supportive supervision of medical students on rural attachments were also explored.^15,16^

Authors from South Africa described the process of developing longitudinal placements of medical students with distributed sites that included regional and district hospitals, community health centres and clinics for varying periods of time.^5,17,18,19^ The purpose of these placements is to integrate medical students into clinical teams in generalist environments, where they can learn from interprofessional relationships and from patients, and appreciate how the referral system works from primary to tertiary care.^18^ Their exposure to interprofessional and multidisciplinary teamwork, providing comprehensive and continuous care, enabled students to become more self-aware and critically reflective as part of their professional identity formation and maturity as trainee health professionals.^5^

These developments evolved from application of core values in curriculum design that were aligned with the values of FM and PC and applicable to FM training programmes in all African countries. These values included understanding the local context, especially societal needs and social determinants of health; developing agency and self-efficacy; interprofessional, intersectoral and peer-based learning, collaboration and teamwork; engagement and accountability to communities served, with commitment to social justice, democracy and equity.^5^ Use of reflective writing, giving and receiving feedback and debriefing were important learning methods referred to in several reports, with assessment of this learning through use of log books, portfolios with evidence of critical reflections and appraisals, project reports and interviews.^5,6,16,19^ Although most of the reports were of medical student training, the same principles can be applied to other health professional training.^18^

The Angola FP training programme, aligned with the Cuban model, is mainly based in community health centres and municipal hospitals, with the aim of covering every municipality in the country.^10^ In most African health systems, there are insufficient numbers of specialist FPs to provide first-contact PC or tutors to supervise their training in PC. Important roles for FPs are therefore as capacity-builders, educators, mentors and supervisors of other cadres who are PC providers such as nurses and clinical officers (COs). Authors described teaching and higher diploma programmes designed by FPs in Kenya and Malawi, which enabled COs to be better skilled at providing frontline PC services across their countries, and to become trainers for the next generation of COs.^20,21^ Emergency simulation drills for multidisciplinary teams at PC facilities, provided by FPs in one district of South Africa, led to improved skills and confidence, teamwork and sharing of information in response to emergencies presenting at PC clinics.^22^

International partnerships such as the Train the Clinical Trainers, between the Royal College of General Practitioners (UK) and the South African Academy of FPs, used workplace-based training and assessment methods to provide clinical trainers with educational skills. The successful implementation of this partnership raised hopes of extending the training to other African countries.^4^ Newer FP training programmes in Ethiopia^11^ and Angola^10^ benefitted from collaboration with more established programmes in partner countries. The partnerships supported regular virtual meetings, in-person faculty exchange visits, curriculum support, faculty development, teaching and leadership training, examination development and certification of trainees. Application of successful training models from established programmes provided credibility and high standards, while ensuring relevance by adaptation to local contexts and a two-way exchange of knowledge and experiences.

Authors noticed that health systems remain heavily focused on the premise that vertical specialist care was superior to generalist PC. This focus stands in contrast to health policies that emphasise commitment to primary health care, which has demonstrated improved health outcomes while reducing costs for users and governments.^8,10,15^ The reports in this special collection show a richness and depth of educational innovations in FM, which deserve greater recognition for their contribution to stronger PC systems. If scaled up with a critical mass of FPs acting as change agents, such educational programmes could radically transform health for populations in Africa.
